# Leptospirosis in the western Indian Ocean islands: what is known so far?

**DOI:** 10.1186/1297-9716-44-80

**Published:** 2013-09-09

**Authors:** Amélie Desvars, Alain Michault, Pascale Bourhy

**Affiliations:** 1 Unité Mixte de Recherche Contrôle des Maladies Animales Exotiques et Emergentes (UMR CMAEE), Centre de Coopération Internationale en Recherche Agronomique pour le Développement (CIRAD), Sainte-Clotilde, La Réunion, France; 2 Laboratoire de Bactériologie-Parasitologie-Virologie-Hygiène, Groupe Hospitalier Sud Réunion (GHSR), Centre Hospitalier Régional (CHR), Saint-Pierre, La Réunion, France; 3 Institut Pasteur, Unité de Biologie des Spirochètes, Centre National de Référence de la Leptospirose, Paris, France

## Abstract

In the past decade, leptospirosis has emerged as a major zoonosis with a worldwide distribution. The disease is caused by bacteria of the genus *Leptospira*. The western Indian Ocean includes more than one hundred tropical or subequatorial islands where leptospirosis constitutes a major public health problem. The clinical signs of the human disease are generally similar to an influenza-like syndrome, but acute forms of the disease are reported and mortality remains significant in this region. In animals, clinical forms are mainly asymptomatic but leptospirosis reduces the fertility of livestock, resulting in economic losses. The data available about human and animal leptospirosis in the western Indian Ocean islands are diverse: human leptospirosis has been extensively studied in Reunion Island, Mayotte, and the Seychelles, whereas the human clinical disease has never been described in Madagascar, Comoros, Mauritius, or Rodrigues, mainly because of the deficiency in appropriate medical and diagnostic structures. The rat is recognized as the major reservoir host for the bacteria on all islands, but recent data from Reunion Island indicates that almost all mammals can be a source of contamination. The incidence of leptospirosis in humans is highly seasonal, and linked to the rainy season, which is favorable for the environmental maintenance and transmission of the bacteria. The epidemiology of leptospirosis is fully island-dependent, related to the number of mammalian species, the origins of the introduced mammalian species, the relationships between humans and fauna, and environmental as well as cultural and socio-economic factors.

## Table of contents

1. Introduction

2. Regional context

3. Known *Leptospira* hosts in the western Indian Ocean islands

  3.1 Animal hosts

  3.2 Human leptospirosis in the western Indian Ocean islands

4. Clinical presentation of leptospirosis and epidemiological risk factors in the western Indian Ocean islands

  4.1 Clinical presentations

  4.2 Risk factors of contamination in animals and humans

5. Molecular epidemiology and genetic characterization of circulating strains

6. Conclusions

7. Abbreviations

8. Competing interests

9. Authors’ contributions

10.  Acknowledgements

11.  References

## 1. Introduction

Leptospirosis, a bacterial disease caused by pathogenic species from the genus *Leptospira* (phylum Spirochaetes), is probably the most widespread zoonotic disease in the world [1] and is most commonly found in tropical and subtropical countries [2,3]. Leptospirosis is maintained by the persistent colonization of the renal tubules of carrier animals, and it appears that almost all mammals are susceptible to be natural carriers of *Leptospira*[4-6]. An infected animal can remain symptom-free and shed infectious organisms in its urine, either transitorily or for its entire lifetime [5,7]. Humans can be infected directly by contact with the urine of an infected animal or indirectly from the contaminated environment [5]. The survival of the bacterium outside the host generally requires humid and warm conditions [7]. The genus *Leptospira* comprised the saprophytic subgroup (with six known species), the pathogenic subgroup (nine species), and the intermediate subgroup (five species) the pathogenicity of which remains unclear [8]. The pathogenic species comprise more than 250 serovars belonging to approximately 24 serogroups based on agglutinating lipopolysaccharide antigens [8]. This serological classification is widely used in veterinary and human epidemiological studies but remains incompatible with more modern molecular classification (Table 1) [5]. The most widely used test is the microscopic agglutination test (MAT) in which patient sera are mixed with antigen suspensions of live *Leptospira* and examined by dark-field microscopy for agglutination [5]. This test, on which much of our data is based, suffers from several limitations (reproducibility, restricted panels of antigens, lack of sensitivity, and specificity), and a comparison between different studies conducted in different places is currently not possible [9-11]. Genetic characterization of isolates involves various genomic methods, such as sequencing of 16S rRNA gene, pulsed-field gel electrophoresis (PFGE), multi-locus sequence typing (MLST), or multiple-loci variable number tandem repeat (VNTR) analysis (MLVA) [8].

**Table 1:** **Distribution of the serogroups cited in the text within *Leptospira *genomospecies.**

**Genomospecies**	**Serogroups**
*L. interrogans*	Icterohaemorrhagiae, Canicola, Pomona, Australis, Autumnalis, Pyrogenes, Grippotyphosa, Sejroe, Mini
*L. borgpetersenii*	Hebdomadis, Tarassovi, Australis, Autumnalis, Pyrogenes, Sejroe, Mini, Ballum
*L. kirschneri*	Icterohaemorrhagiae, Canicola, Pomona, Australis, Autumnalis, Grippotyphosa
*L. noguchii*	Australis, Autumnalis, Pyrogenes, Pomona
*L. fainei*	Hurstbridge

The table shows the serogroups most often found in human clinical cases and in animals by epidemiological surveys (from [5]).

The western Indian Ocean islands are closed ecological ecosystems where the biocenosis involves both endemic and introduced host species and pathogens. This “composite” biodiversity [12], and the particularities of local ecological conditions as well as agricultural and cultural practices, result in divergences in the epidemiology of the disease between islands [13]. Data about animal leptospirosis in the western Indian Ocean islands remain scarce. This review is aimed at describing the diversity and the distribution of the pathogenic leptospiral serovars/serogroups as well as the epidemiological features of human and animal leptospirosis in some islands of the western Indian Ocean area. Data on leptospirosis have not been published or are not available for all of the islands in this region, and consequently the review addresses mainly the islands of Madagascar, the Seychelles, Mayotte, and Reunion.

## 2. Regional context

**Figure 1:**
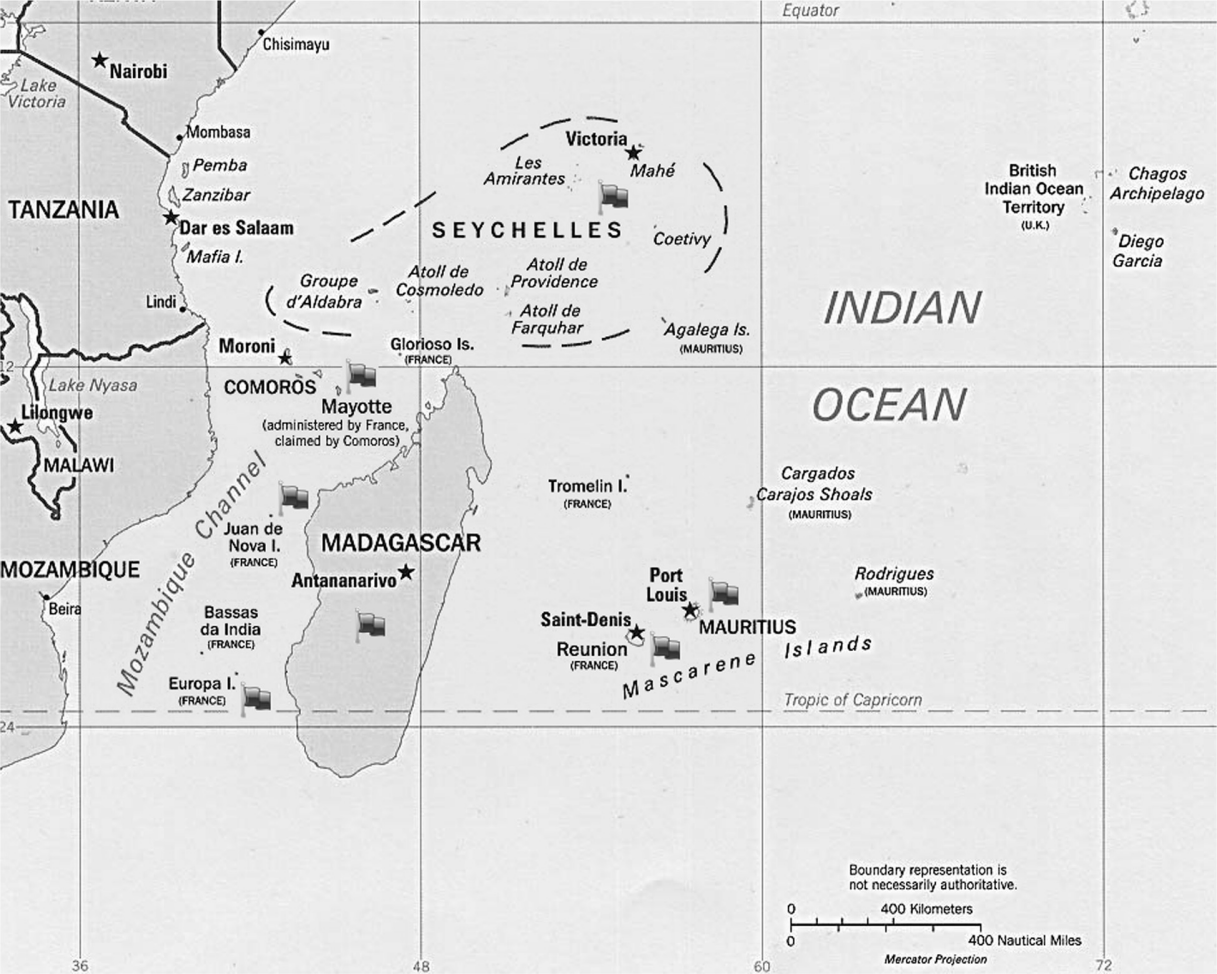
**Map of the western Indian Ocean region.** Gray flags indicate islands for which data on leptospirosis is available.

The western Indian Ocean counts 21 inhabited groups of islands belonging to 12 countries (Figure 1). The terrestrial surface areas of the western Indian Ocean islands are between < 1 km^2^ (Bassas da India) and 587 041 km^2^ (Madagascar). Most islands of this region have a tropical climate with two main seasons: a hot and rainy season (“austral summer”), and a dry season (“austral winter”). In the Seychelles, the climate is subequatorial with more than 80% of humidity all year round.

## 3. Known *Leptospira* hosts in the western Indian Ocean islands

### 3.1. Animal hosts

Mammalian biodiversity is poor in the volcanic islands of the western Indian Ocean and all mammals currently present, except bats, have been introduced. However, Madagascar is one of the most striking hotspots of biodiversity on Earth where most of the fauna is endemic [14]. Table 2 summarizes the various *Leptospira* hosts studied in the western Indian Ocean region. Leptospirosis is probably endemic in the mammalian species of the western Indian Ocean islands Table 2[15-17]. In Mayotte and Reunion Island, serological surveys showed a high seroprevalence of leptospirosis in non-vaccinated (stray and domestic) dogs [15,17] and dogs have been demonstrated to be renal carriers and urinary shedders of *Leptospira* on the Reunion Island [17]. In Reunion, the seroprevalence of the disease in dogs has not varied substantially since 1980, and the Canicola serogroup has been described as the main serogroup infecting dogs; Icterohaemorrhagiae is the second most frequent in dogs [17,18]. By contrast, in Mayotte, the serogroup Mini (the main serogroup implicated in human cases) has most frequently been identified by serology in stray and domestic non-vaccinated dogs [15].

**Table 2:** **Potential hosts of *Leptospira *in the western Indian Ocean islands.**

**Species**	**Geographic location**	**Sensitive**^**(1) **^**(S) or resistant**^**(2) **^**(R)**	**Main clinical signs**	**Asymptomatic chronic shedding**	**Economic significance**	**Existing data on leptospirosis in the Indian Ocean region**
**Primates**						
Humans	All islands	S (asymptomatic forms are frequent)	Fever, myalgias, headache, chills, oliguria/anuria, jaundice, conjunctive suffusions, aseptic meningitis, hemorrhages, skin rash, renal and hepatic failure, severe pulmonary hemorrhagic syndrome	Yes	Medical costs, absence from work, mortality	[19-35]
Brown lemur *(Eulemur fulvus)*	Mayotte, Madagascar	Nd	Nd	Nd	Nd	[15]
Crab-eating macaque *(Macaca fascicularis)*	Mauritius	Nd	Nd	Nd	Nd	No
Mongoose lemur *(Eulemur mongoz)*	Comoros, Madagascar	Nd	Nd	Nd	Nd	No
**Rodents**						
Black rat *(Rattus rattus)*	All islands	R	Absent	Yes	Cost of control (traps, poison, time)	[15,17,36,37]
Norway rat *(R. norvegicus)*	Reunion, Mauritius, Seychelles, Madagascar	R	Absent	Yes	Cost of control (traps, poison, time)	[17,36]
Domestic mouse *(Mus musculus)*	All islands	R	Absent	Yes	Cost of control (traps, poison, time)	[17,36]
**Terrestrial insectivores**						
Shrew *(Suncus murinus)*	Mauritius, Reunion, Comoros, Madagascar	R	Absent	Yes	Cost of control (traps, poison, time)	[17,36]
Tenrec *(Tenrec ecaudatus)*	Reunion, Mayotte, Comoros, Mauritius, Seychelles, Madagascar	Nd	Nd	Not proved	Nd	[17,38]
**Carnivores**						
Small Indian civet *(Viverricula indica)*	Mayotte, Madagascar	Nd	Nd	Nd	Nd	[37]
Dog*(Canis lupus familiaris)*	All islands	R or S*	Fever, oliguria/anuria, jaundice, haemorrhages, renal and hepatic failure	Yes	Cost of control of the stray populations	[15,17,19]
Cat *(Felis catus)*	All islands	R or S*	Mild, non-specific signs	Yes	Cost of control of the stray populations	[17]
**Livestock**						
Cattle *(Bos taurus)*	All islands	R or S*	Reproductive failure	Yes	Poor reproductive results, abortion, neonatal morbidity	[17,19,20,37,39-41] Pasteur Institute (unpublished data)
Goat *(Capra hircus)*	All islands	R or S*	Reproductive failure	Yes	Poor reproductive results, abortion, neonatal morbidity	[17,37]
Swine *(Sus scrofa)*	All islands	R or S*	Reproductive failure	Yes		[17,19,20]
Rusa deer *(Timorensis rusa)*	Reunion, Mauritius	R or S*	Reproductive failure	Yes	Poor reproductive results, abortion, neonatal morbidity	[17]
**Equine**						
Horse *(Equus ferus)*	All islands	R or S*	Acute form, reproductive failure, chronic uveitis	Nd	Cost of veterinary care, abortion, neonatal morbidity	[19,41]
**Bats**						
Free-tailed bat *(Mormopterus francoismoutoui)*	Reunion	Probably R	Nd	Yes	Nd	[17]
Seychelles flying-fox *(Pteropus seychellensis)*	Mayotte, Comoros, Seychelles	Probably R	Nd	Nd	Nd	[15,37]
Peters’s wrinkle-lipped bat *(Mormopterus jugularis)*	Madagascar	Probably R	Nd	Nd	Nd	[42]
Madagascar free-tailed bat *(Otomops madagascariensis)*	Madagascar	Probably R	Nd	Nd	Nd	[42]
Trouessart’s trident bat *(Triaenops furculum)s*	Madagascar	Probably R	Nd	Nd	Nd	[42]
Trident bats *(Triaenops menamena)*	Madagascar	Probably R	Nd	Nd	Nd	[42]
Glen’s long-fingered bat *(Miniopterus gleni)*	Madagascar	Probably R	Nd	Nd	Nd	[42]
*Miniopterus griffithsi*	Madagascar	Probably R	Nd	Nd	Nd	[42]
*Miniopterus mahafaliensis*	Madagascar	Probably R	Nd	Nd	Nd	[42]
Malagasy mouse-eared bat *(Myotis goudoti)*	Madagascar	Probably R	Nd	Nd	Nd	[42]
Comoro rousette *(Rousettus obliviosus)*	Comoros	Probably R	Nd	Nd	Nd	[42]
Western Seychelles Free-tailed bat *(Chaerephon pusillus)*	Mayotte, Comoros	Probably R	Nd	Nd	Nd	[42]
*Miniopterus griveaudi*	Comoros, Madagascar	Probably R	Nd	Nd	Nd	[42]

^(1)^ The animal is infected and shows clinical signs of the disease.

^(2)^ The animal is infected without clinical signs of the disease.

* These species are generally asymptomatic, but some *Leptospira* strains can induce acute forms of the disease (e.g. Icterohaemorrhagiae in dog or in horse).

Nd: no data.

Leptospirosis has been known since 1980 to be a major infectious disease in cattle in Reunion Island [19] and in 2003, a study showed that serogroups Sejroe and Hebdomadis were major causes of abortion in dairy cattle [39]. Sejroe was reported to be the main serogroup circulating in beef and dairy cattle in Reunion Island in 2009 [17], whereas in Mayotte, cattle are mostly infected by serogroup Mini (National Reference Center for Leptospirosis, France, unpublished data). The original Hebdomadis serogroup is divided into three separate serogroups according to their serological affinities: Hebdomadis, Sejroe and Mini [43]. Given the absence of isolation data and the antigens used in serological studies, the seroreactivity to Sejroe, Mini, and Hebdomadis serogroups in cattle could be cross-reactions hiding a serological response to only the serovar Hardjo*,* for which cattle are the maintenance hosts [7].

Bovine and pig leptospirosis has long been suspected in Madagascar [44] but the renal carriage of *Leptospira* has never been documented in these species [40]. Nevertheless seroprevalence in apparently healthy cattle and pigs was reported in 1968 in the south region of the island, with the predominance of the Grippotyphosa serogroup [20]. Desvars et al. [17] reported that in Reunion Island, serogroup Pyrogenes and Panama are the most prevalent serogroups found by MAT in goats, Rusa deer, and pigs whereas they are rarely reported in diagnosed patients [45].

Serological surveys of the insectivorous tenrec *(Tenrec ecaudatus)* in Reunion Island report seroprevalences of between 13.2% (5/38) [17] and 92% (34/37) [38] in this species, probably depending on the geographical area of sampling and/or age of the animals. *Leptospira* could not be evidenced in tenrec kidney or urine, suggesting that this species is probably not a chronic reservoir host for the disease [17].

The epidemiological role of bats in the transmission of *Leptospira* attracts more and more scientific interest [46-53]. In Madagascar, antibodies to *Leptospira* could not be evidenced in the fruit bat *Pteropus rufus*[54,55], but recently, pathogenic *Leptospira* spp. were found in bats, in Madagascar and Union of Comoros [42]. In Mayotte, a recent study reported a seroprevalence of 10.2% (5/49) in *Pteropus seychellensis* with Pyrogenes and Grippotyphosa as infecting serogroups [15]. The free-tailed bat *(Mormopterus francoismoutoui*, family Molossidae) is a urinary shedder of *Leptospira*[17]. Nevertheless, the zoonotic role of bats species in the transmission of leptospirosis to humans remains uncertain.

Serological evidence of leptospirosis has been reported in lemurs from Mayotte but at a low seropositive rate (2%, 1/50) [15]. This low rate is probably due to the arboreal lifestyle of these animals minimizing their contact with contaminated water or soil. Sensitivity to leptospirosis differs greatly between non-human primates [56-59]. We could also hypothesize that lemurs are highly sensitive to leptospirosis such that infected animals die (as demonstrated in the squirrel monkey, *Saimiri sciureus,* and the marmoset, *Callithrix jacchus*[56,58,59]). Thus the contribution, if any, of primates in the transmission of leptospirosis in Mayotte, Anjouan, Moheli, and Mauritius still needs to be elucidated.

All published studies confirm that the black rat is the major reservoir host for *Leptospira* in Europa and Juan de Nova [60], Mayotte (where the Norway rat is absent) [15], Reunion Island [17], and Madagascar [36]. In Reunion Island and Madagascar, the Norway rat*,* the shrew, and the domestic mouse have also be shown to be renal carriers and/or urinary shedders of *Leptospira*[17,36]. In Reunion Island, the major serogroup identified in the rat is Icterohaemorrhagiae, but other serogroups also seem to circulate (Canicola, Sejroe) [17]. In Mayotte, the Mini serogroup is the main circulating serogroup in *R. rattus* and there is strong evidence that the black rat population is the major reservoir of *Leptospira* and source of its transmission to humans [15].

### 3.2. Human leptospirosis in the western Indian Ocean islands

Acute leptospirosis has never been described in Madagascar and investigations conducted locally have failed to show the presence of the bacterium in humans. Lhuiller et al. [55] reported a low seroprevalence rate among Antananarivo inhabitants. The only autochtonous clinical case (identified in the 1950’s) to be confirmed serologically had antibodies to serogroup Australis [54,61]. In 1968, Silvérie et al. [20] identified that Tarassovi, Grippotyphosa, Australis, and Hebdomadis were the most prevalent serogroups in the human population in the region of Toliara. They reported a seroprevalence of 50.8% (33/65) [20] which seems surprisingly high in a country where no clinical cases have been reported. Recently, one human case was diagnosed at the hospital of Mamoudzou (Mayotte) involving *Leptospira kirschneri* serogroup Mini and was suspected to have been imported from Madagascar [21].

The first confirmed case of human leptospirosis in the Maldives was reported in November 2000. Since then, the disease has been under national surveillance [62].

The disease has been very occasionally reported in Mauritius, but there is probably underreporting, since the epidemiological conditions are very similar to those in the neighboring Reunion Island [63]. Simon et al. [22] recently described a case of leptospirosis in a French patient who had traveled to Mauritius.

The Seychelles presents the highest incidence of leptospirosis in the western Indian Ocean area and one of the highest incidences in the world [23]. Between 1988 and 1990, the annual incidence was 60 cases per 100 000 inhabitants and serogroups Icterohaemorrhagiae and Autumnalis were most frequently identified during this period [64]. In 1995–1996, the incidence of leptospirosis was estimated to be 101 per 100 000 [23] and eight serogroups were identified, with Icterohaemorrhagiae and Hurstbridge (the latter commonly considered as non-pathogenic) being the main circulating serogroups.

The first publication describing human leptospirosis in Mayotte was in 1990 [24]. It reported 42 cases between 1984 and 1989 and an annual incidence of 3.83 per 100 000 [24]. Recent efforts in the detection of the disease [21,25] has led to the annual incidence of leptospirosis being re-evaluated, as 25 per 100 000. Mini is the major serogroup responsible for human clinical cases in Mayotte whereas Icterohaemorrhagiae has never been isolated from patients in Mayotte which represents a unique epidemiological situation [21]; the other *Leptospira* serogroups identified in patients are Pyrogenes, Grippotyphosa, and Pomona.

In Reunion Island, the annual number of human cases of leptospirosis has varied little since 1970, with an average of 40 cases per year between 1970 and 1979 and an average of 55.7 cases between 1998 and 2008 [65] (probably due to improved diagnosis of this disease). The incidence of leptospirosis was 15.13 per 100 000 in 2010 [45]. Between the 1970s and now, serogroup Icterohaemorrhagiae has been the main serogroup involved in clinical cases on Reunion Island [26-28,45,65].

## 4. Clinical presentation of leptospirosis and epidemiological risk factors in the western Indian Ocean islands

### 4.1. Clinical presentations

In humans, clinical leptospirosis has protean manifestations but generally causes a febrile illness that often, in its early phase, cannot be differentiated from other acute influenza-like fevers such as dengue, influenza, chikungunya, or malaria [5,8] (Table 2). In the islands of the western Indian Ocean where human leptospirosis is found, less than 10% of hospitalized leptospirosis patients die [21,29,66]. In the Seychelles, about one-third of cases of leptospirosis are mild forms; two-thirds have a more severe presentation with jaundice (without liver failure) and/or acute renal failure and/or pulmonary hemorrhage [23]. In the Seychelles, during a 12-month period in 1995–1996, 8% (6/75) of the patients with acute leptospirosis died [29]; autopsies showed that diffuse bilateral pulmonary haemorrhage was the main cause of death [29]. In Reunion Island, clinical forms of the disease in hospitalized patients are often severe [26] and pulmonary manifestations are frequent [28,67-69]. In Mayotte, 3.2% of the hospitalized cases are fatal [21].

Leptospirosis in livestock is generally associated with reproductive failure (Table 2) [70-72] but the studies published to date do not permit evaluation of the true losses caused by the disease in dairy and beef cattle, pigs, goats, sheep, and Rusa deer in the different islands. Economic data of this type may encourage taking this disease into account in the management of reproduction and health problems within herds. In dogs, leptospirosis can, in some cases, cause an acute disease similar to Weil disease in humans, but mostly, the disease is asymptomatic in dogs (Table 2) [73].

### 4.2. Risk factors of contamination in animals and humans

The risk factors for acquiring leptospirosis in animals have not been studied in the western Indian Ocean region. Nevertheless, risk factors for contamination of both individual animals and herds are most probably similar to those identified in other tropical countries. [74-79].

In humans from Reunion Island and Mayotte, the risk of contamination is the highest for the 20 to 40 year-old age group [21,26], and in the Seychelles, the mean age of cases is 34 years (and 53 years in fatal cases) [23]. Risk factors for acquiring leptospirosis are similar in all tropical islands. One common feature of the disease is that leptospirosis is more frequent among men than women [21,23,28]. This sex difference, is usually attributed to occupational and behavioral factors [13,80]. In the Seychelles, significant associations have been found between leptospirosis cases and activities in the forest, gardening, and refuse not collected by public services (which favors high rat population density) [23,30]. Washing clothes or bathing in the river, and walking barefoot, favor contamination by *Leptospira*[23,30] and an association between the disease and wet soil around homes has been reported in the Seychelles [23] and Reunion Island [28].

**Figure 2:**
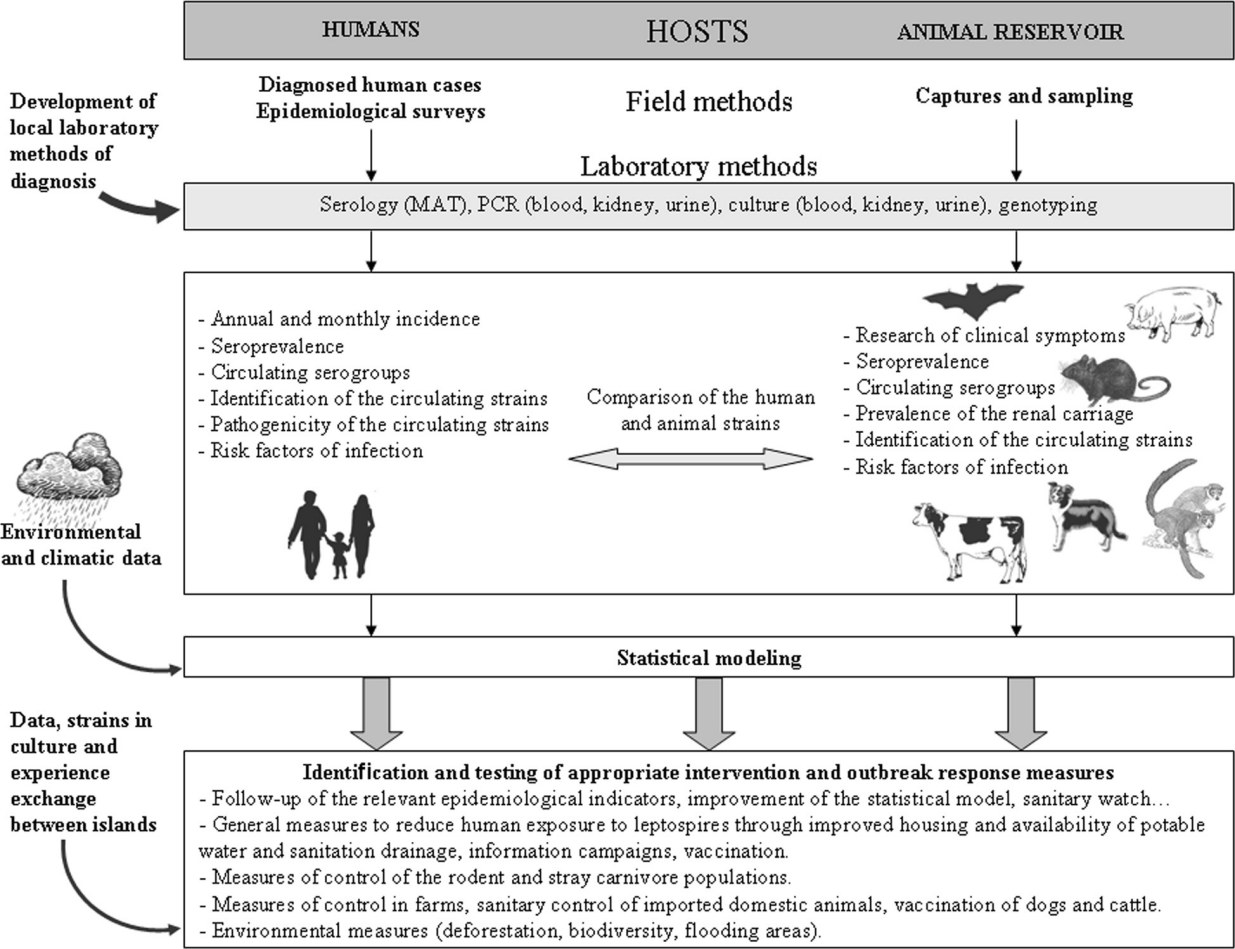
**Global methods of investigation of leptospirosis.** Islands are small (except Madagascar) closed territories in which the number of mammal species is known (except in Madagascar) and each can be studied. Transdisciplinary approaches, incorporating diverse disciplines and approaches specific to leptospirosis should contribute to a better understanding of the mechanisms of transmission in the different ecosystems across the region. Interaction and data exchange between the various research teams of the western Indian Ocean islands is crucial.

The incidence of leptospirosis in humans is affected by rainfall, particularly in tropical regions [81-83]. In Reunion Island, cases of leptospirosis are reported all year round, but epidemics mostly occur during the rainy season [26,28,31,84]. Therefore, despite popular belief, the peak incidence of leptospirosis in Reunion Island is not during the sugar cane harvest (July to December) [84]. This seasonality in leptospirosis cases is also observed in Mayotte where almost all cases are reported during the rainy season [21]. In the Seychelles, the relationship between rainfall and leptospirosis cases is weaker than in Mayotte or Reunion Island, probably because the rainy season is not well-defined [30]. A prediction model could be a useful tool for the identification of cases and, obviously, for predicting epidemics; this may facilitate improved diagnosis and treatment of leptospirosis to reduce lethality rates [85,86]. Statistical modeling should include the number of cases, meteorological factors, and data on animal and human population density (Figure 2).

## 5. Molecular epidemiology and genetic characterization of circulating strains

Genetic and serological characterization of *Leptospira* isolates requires considerable effort in the field with successful cultures from clinical specimens. *Leptospira* strains from various islands of the western Indian Ocean have been characterized, notably the Reunion Island, Mayotte and Madagascar. A clinical isolate from the Reunion Island was identified as *L. interrogans* serogroup Icterohaemorrhagiae (Pasteur Institute, Paris, and GHSR, unpublished data). Since 2007, more than one hundred *Leptospira* strains have been isolated from patient blood samples on Mayotte [21], and sequencing and MLVA, have classified these human isolates into four genomospecies: *L. interrogans, L. kirschneri, L. borgpetersenii*, and *L. borgpetersenii group B*, which is a newly described species [21]. Serological typing of these isolates showed that these four species are distributed into four serogroups: Mini, which represents the great majority of the infecting strains, Grippotyphosa, Pomona, and Pyrogenes. All are pathogenic to humans [21,25]. Sequencing of a segment of the16S rRNA gene in *Leptospira* detected in rat kidneys showed that the four pathogenic genomospecies responsible for human cases are also found in rats [15,21]. The genetic diversity of *Leptospira* in the rat population is generally low in various locations [87-90] but in Mayotte, rats carry four *Leptospira* genomospecies, making the genetic diversity of *Leptospira* strains infecting black rats on this island unique [15].

The ten isolates obtained in 2008–2009 in Madagascar from *R. rattus* and *R. norvegicus* kidneys were all identified by PFGE and MLVA as *L. interrogans* serogroup Canicola serovar Kuwait. This was the first isolation of a *Leptospira* strain on this large island [36]. The strain isolated from a patient in Mayotte returning from Nosy Be (Madagascar) was genetically closely related to clinical *Leptospira* isolates from Mayotte, but the MLST pattern was different, indicating that this strain did not originate from Mayotte but most probably from Madagascar [21]. Recently, the sequencing of seven fragments of the 16S rRNA gene from *Leptospira* detected in bats from Comoros islands and Madagascar showed that three were closely related to *L. borgpetersenii*, one grouped with *L. interrogans*, and three were not associated with any described species [42]. Our studies suggest that *Leptospira* from Mayotte and Madagascar are closely related at the genome level, which is in agreement with previous studies [21,42]. Phylogeographic patterns support the hypothesis of a human-mediated colonization of *R. rattus* from source populations of India and the Arabian Peninsula to islands of the western Indian Ocean [91]. Independent colonization events may have occurred simultaneously in Madagascar and Grande Comore, whereas rats from Mayotte were introduced from Madagascar [91]. If *Leptospira* strains are introduced in a new geographical area *via* their hosts (in the western Indian Ocean islands, they have most probably been introduced with their preferential host *Rattus* sp by boat) [92], thus *Leptospira* strains identified in Mayotte are probably mainly derived from strains of Madagascar.

It would be beneficial to establish a consensus on genotyping methods, at least on the regional scale, to facilitate comparison of the circulating strains and the epidemiology of this major infectious disease. This may help the implementation of adapted island-specific and cost-effective preventive measures.

## 6. Conclusions

Leptospirosis seems endemic to all the human- and animal-inhabited islands of the western Indian Ocean region. It is supposed that *Leptospira* is introduced onto islands with their animal host, and that a variable number of introduced strains have adapted to the new local environment and available hosts [92]. The genetic diversity of the insular black rat populations, which have been introduced from different geographic areas (Europe, east Africa) [91] associated with the local and isolated co-evolution of *Leptospira* and this preferential host may explain the island-specificity of the circulating strains [15,17]. Moreover, the genetic biodiversity of leptospires in a closed range, such as an island, is also affected by geography, soil, climate, biotic interactions, and anthropogenic activities [6,93]. The mammalian diversity in Madagascar provides a wide range of potential hosts for *Leptospira*; however, the substantial haplotype diversity of the black rat population of Madagascar [91] favors co-evolution between *Leptospira* and several genetically different preferential hosts. Moreover, Madagascar also has a wide diversity of ecosystems. In view of these various factors, this island presents optimal conditions for genetic diversification of the genus *Leptospira*, as a consequence of environmental pressure and host-adaptation.

An effective surveillance system is essential for preventing and controlling outbreaks of leptospirosis [94]. Nonetheless, this review illustrates how knowledge about this zoonosis differs significantly among islands. In the Seychelles, human leptospirosis is probably the major known health problem in the country, but nevertheless, the animal reservoir has not been sufficiently studied.

The prevalence of the different *Leptospira* serogroups in a human population depends strongly on the local reservoir hosts and the strains they carry [6]. Consequently, knowledge of animal leptospirosis is essential for a better understanding of the disease in humans (Figure 2). On islands, mammalian biodiversity is closely linked with the incidence of human leptospirosis cases [12]. Since leptospirosis is a transdisciplinary problem [95], studies on the animal reservoir could be extremely useful where the epidemiology of the human disease and the human strains is well described, and should be done in parallel with collecting climatic and socio-environmental data [95]. Predictions of global warming and an increasing frequency and severity of cyclones in the Indian Ocean due to global climate change, suggest an increased risk of flooding, and exacerbation of the disease burden from leptospirosis [1,96-100].

Studies on leptospirosis are generally conducted independently, by different research teams, and the methods of investigation and available data vary greatly between islands. As a result, studies are generally not comparable with each other, leading to a “patchwork” of isolated data. We believe that it would be beneficial to standardize the methods of diagnosis of leptospirosis, at least, throughout the western Indian Ocean region: the same panel of antigens could be used for MAT and the same primers for PCR-based investigations. The methods of typing also need to be standardized across the region to allow genetic comparison of the strains, and a regional collection of isolates would be useful (Figure 2). This will necessitate pooling resources for a better knowledge of the disease and for improving the cost-effectiveness and efficacy of preventive measures.

## Abbreviations

GHSR: Groupe hospitalier sud Réunion; MAT: Microscopic agglutination test; MLVA: Multiple-loci variable number tandem repeat analysis; MLST: Multi-locus sequence typing; PCR: Polymerase-chain reaction; PFGE: Pulsed-field gel electrophoresis; VNTR: Variable number tandem repeat; 16S rRNA: 16S ribosomal ribonucleic acid.

## Competing interests

The authors declare that they have no competing interests.

## Authors’ contributions

AD and PB performed a study on the literature available on the subject, analyzed the retrieved information and wrote the manuscript. AM revised the manuscript critically according to their areas of expertise. All authors read and approved the final manuscript.
